# Genetic Transformation of Sugarcane, Current Status and Future Prospects

**DOI:** 10.3389/fpls.2021.768609

**Published:** 2021-11-11

**Authors:** Florencia Budeguer, Ramón Enrique, María Francisca Perera, Josefina Racedo, Atilio Pedro Castagnaro, Aldo Sergio Noguera, Bjorn Welin

**Affiliations:** ^1^Instituto de Tecnología Agroindustrial del Noroeste Argentino (ITANOA), Estación Experimental Agroindustrial Obispo Colombres (EEAOC) – Consejo Nacional de Investigaciones Científicas y Técnicas (CONICET), Las Talitas, Argentina; ^2^Centro Cientifico Tecnológico (CCT) CONICET NOA Sur, San Miguel de Tucumán, Argentina

**Keywords:** disease resistance, drought tolerance, herbicide resistance, genome editing, pest resistance, *Saccharum* hybrids, transformation methods

## Abstract

Sugarcane (*Saccharum* spp.) is a tropical and sub-tropical, vegetative-propagated crop that contributes to approximately 80% of the sugar and 40% of the world’s biofuel production. Modern sugarcane cultivars are highly polyploid and aneuploid hybrids with extremely large genomes (>10 Gigabases), that have originated from artificial crosses between the two species, *Saccharum officinarum* and *S*. *spontaneum*. The genetic complexity and low fertility of sugarcane under natural growing conditions make traditional breeding improvement extremely laborious, costly and time-consuming. This, together with its vegetative propagation, which allows for stable transfer and multiplication of transgenes, make sugarcane a good candidate for crop improvement through genetic engineering. Genetic transformation has the potential to improve economically important properties in sugarcane as well as diversify sugarcane beyond traditional applications, such as sucrose production. Traits such as herbicide, disease and insect resistance, improved tolerance to cold, salt and drought and accumulation of sugar and biomass have been some of the areas of interest as far as the application of transgenic sugarcane is concerned. Although there have been much interest in developing transgenic sugarcane there are only three officially approved varieties for commercialization, all of them expressing insect-resistance and recently released in Brazil. Since the early 1990’s, different genetic transformation systems have been successfully developed in sugarcane, including electroporation, *Agrobacterium tumefaciens* and biobalistics. However, genetic transformation of sugarcane is a very laborious process, which relies heavily on intensive and sophisticated tissue culture and plant generation procedures that must be optimized for each new genotype to be transformed. Therefore, it remains a great technical challenge to develop an efficient transformation protocol for any sugarcane variety that has not been previously transformed. Additionally, once a transgenic event is obtained, molecular studies required for a commercial release by regulatory authorities, which include transgene insertion site, number of transgenes and gene expression levels, are all hindered by the genomic complexity and the lack of a complete sequenced reference genome for this crop. The objective of this review is to summarize current techniques and state of the art in sugarcane transformation and provide information on existing and future sugarcane improvement by genetic engineering.

## Introduction

Sugarcane is an important tropical and sub-tropical vegetatively propagated crop cultivated on nearly 27 million hectares in more than 120 countries around the globe, which contributes to more than 75% of the world’s total sugar production (Aslam et al., 2018). In addition to traditional sugar production, sugarcane is recognized as an important energy and biofuel crop due to its great biomass production and large-scale molasses-based ethanol production. Furthermore, it is the most efficient feedstock for the generation of bio-butanol and diesel, and is responsible for 40% of the world’s total biofuel production. Other valuable by-products obtained from sugarcane production are paper, acetic acid, plywood and industrial enzymes among others (Rahman et al., 2019).

Modern sugarcane cultivars are hybrids derived from interspecific crossings between *Saccharum officinarum* (2n = 80), the noble sugar-producing species, and the wild species *S. spontaneum* (2n = 40 − 128) with high fiber content and stress tolerance (Piperidis et al., 2010; Vieira et al., 2018). These hybrids possess highly polyploid and aneuploidy genomes of 53–143 chromosomes (Ingelbrecht et al., 1999), with an estimated size of >10 gigabases (Gb) (Zhang et al., 2012). Genetic studies have shown that the alloautopolyploid genome of these hybrids contains about 80% of chromosomes from *S. officinarum*, 10% of chromosomes from *S. spontaneum*, and 10% recombinant chromosomes between the two progenitor species (D’Hont et al., 1996). To better understand the complexity of the sugarcane genome several initiatives had led to different sequencing projects around the world of both parental species as well as hybrids genotypes (Garsmeur et al., 2018; Zhang et al., 2018; Souza et al., 2019)^1^.

There are many conventional sugarcane breeding programs at different research institutes that are constantly trying to develop new hybrid varieties with higher yields and increased sugar contents, to comply with the increasing pressure to enhance productivity and to sustain profitable sugar industries (Tiwari et al., 2010). However, traditional breeding of sugarcane is very costly, extremely laborious and time-consuming (it takes 10–15 years to release a new elite variety) as a consequence of the genetic complexity, its narrow genetic base, the slow breeding gain, and its susceptibility to various important diseases and pests. These breeding aspects, together with its vegetative propagation, make sugarcane an excellent candidate for crop improvement through genetic engineering (Ingelbrecht et al., 1999). For that reason, serious efforts to improve sugarcane crops by genetic transformation approaches have been carried out during the last three decades (Tiwari et al., 2010) in many sugarcane producing countries.

## Transformation Methods

Two major scientific breakthroughs in the early eighties marked the beginning of genetic transformation of plants and the initiation of a third green revolution (Olmedo, 1999). The first one was the discovery of the ability of *Agrobacterium tumefaciens* to integrate a fragment of its own DNA into the genome of a plant and expressing new proteins in the transformed cell (Herrera-Estrella et al., 1983). This significant event was quickly followed by the report of a direct delivery system by shooting foreign DNA into plant cells known as “biolistic” in order to circumvent some inherent limitations of the biological method (Klein et al., 1987). Both methods allowed genetic engineering to be quickly adopted as a useful tool for breeding programs in many different crops since it allows the incorporation of a single characteristic into elite varieties, retaining their valuable agronomical characteristics (Joyce et al., 1998). This was also true for sugarcane where the first successful reports during the 1990’s about generation of transgenic sugarcane plants were published (Bower and Birch, 1992; Bower et al., 1996; Gallo-Meagher and Irvine, 1996; Arencibia et al., 1998).

A successful gene transfer system for plants requires the integration of: tissue culture to obtain cells competent for genetic transformation and subsequent whole plant regeneration, an efficient gene transfer system to deliver the DNA into the plant genome and an efficient selection system allowing for the identification of transformed cells (Birch, 1997). Even though a wide range of procedures including *A. tumefaciens*, biobalistic, electroporation and polyethylene glycol, by using protoplasts, leaf rolls, or embryogenic callus as explant have been proposed to incorporate genes into sugarcane, biobalistic or *A. tumefaciens* on embryogenic callus or leaf rolls are by far the most widespread approaches (Mohan et al., 2020) and will be further described below.

The first transgenic sugarcane plant was obtained by Bower and Birch (1992) who described a simple and efficient system of microprojectile bombardment of embryogenic callus, obtaining transgenic plants with selectable genes. This procedure was optimized during the following years (Bower et al., 1996) and transgenic plants with a commercial trait (herbicide resistance) were obtained (Gallo-Meagher and Irvine, 1996). The efficiency of producing transgenic sugarcane through biobalistic transformation is evident from the numerous examples of transgenic sugarcane expressing traits of commercial interest that are found in the literature (see following sections of this review) generated by this method. The success of this transformation technique relies on certain advantages such as rapid gene transfer with high efficacy to specific/non-specific tissues, no host limitation and no vector requirement (Mohan, 2017). The main disadvantages are that it presents a high probability of integration of multiple copies of the transgene in the genome and a very long and complicated tissue culture procedure, which can lead to genomic alterations, genetic rearrangements or transgene silencing. Major factors affecting the outcome of generating transgenic events with particle bombardment are nature of explants, DNA concentration and quality, gold/tungsten particle size, pressure level and distance between the bombardment and target tissue. A key recommendation is to produce numerous individual transgenic events (>50) by using lower DNA concentrations in the transformation process, which often permit finding events that meet the requirements to be eligible for commercial purpose.

With regards to sugarcane transformation using *A. tumefaciens*, the first successful event was reported by Arencibia et al. (1998), who optimized this biological system for sugarcane using the reporter gene *gus*A. Shortly afterward, Enríquez-Obregón et al. (1998) reported the generation of glufosinate-resistant sugarcane generated by *A. tumefaciens*-mediated transformation of the *bar* gene encoding a bacterial PPT acetyltransferase which confers resistance to the broad-spectrum herbicide glufosinate ammonium. However, successful studies using the *A. tumefaciens* system where relatively few initially, which was probably due to several restrictions by employing this method, especially a strong dependence on the genotype and co-cultivation conditions (explant, strains, target gene, culture conditions, media). Notwithstanding, this method theoretically offers important advantages for commercial purpose such as reduced copy number and presents fewer problems with transgene co-suppression and instability (Enríquez-Obregón et al., 1998). Therefore, more effort to improve this method was carried out and from the second decade of the 2000s, various transgenic sugarcane events have been obtained using *A. tumefaciens*-mediated transformation (see Mohan et al., 2020).

Basically, before establishing which of the two methods to be used in a genetic transformation experiment in sugarcane, it is important to know if a genotype is susceptible to *A. tumefaciens* transformation and to examine the transformation efficiency of both methods. However, different transformation efficiencies have been reported arbitrarily defined by different authors, or even the same authors, publishing different definitions for this parameter (Table 1). Therefore, to be able to directly compare transformation frequencies among studies, a unified parameter of the efficiency should be established. Such a parameter should consider the amount of plant material used as explants (input) and the total of transgenic plants (independent events) regenerated after the selection stage (output). Evaluating only number of regenerated plants after the selection procedure is not a very reliable source for determining transformation efficiency, since a high selection pressure will give an underestimation while a less strict selection will permit false positive survival. Another drawback is that not all transformed cells express the selection gene and are therefore not withstanding the selection pressure although being correctly transformed. Nevertheless, it is a very useful parameter for comparison between methods and gives valuable information to decide which one to employ for a specific genotype. To correctly be able to define if a selected plant or tissue has been transformed, it is necessary to perform reliable experimental methods, such as Southern blot hybridization (Birch, 1997). However, considering the complexity of employing this technique to analyze hundreds of putative transformed events, transgene-positive PCR events should be considered as a preliminary screening of the number of events obtained.

**TABLE 1:** Transformation efficiency and copy numbers determined by Southern blot analysis for biolistic and *Agrobacterium*-methods in sugarcane during the last decade.

Transformation method	Variety	Selection gene	Southern blot		Efficiency	Efficiency defined as	References
			N° of plant analyzed	Transgene N° copy (min-max)			
Biolistic	ROC16 and YT79-177	*aph*A	6	6–8	50%	N° of PCR positive regenerates plants	Weng et al., 2011
Biolistic	CP-88-1762	*npt*II	8	1–5	0.57	Transgenic lines per shot	Taparia et al., 2012a
Biolistic	CP 88-1762	*npt*II	48	1–4	1.6	N° of transgenic plant expressing NPTII	Taparia et al., 2012b
Biolistic	Q117	*apha*II	15	1–6	3	Independent resistant plant lines/g fresh weight of bombarded callus	Jackson et al., 2013
*Agrobacterium*	Q117	*apha*II	4	1–4	1	Independent resistant plant lines/g fresh weight of callus	Jackson et al., 2013
*Agrobacterium*	Co 62175, Co 6304, Co 8021, Co 86032, and Co 6907	*bar*	146	1–3	32.6%	N° of GUS positive plants/total n° of sugarcane setts infected * 100	Mayavan et al., 2015
Biolistic	RA 87-3	*npt*II	4	6–9	2.1	PCR positive transgenic plants/bombardment callus	Noguera et al., 2015
*Agrobacterium*	ROC22	*bar*	7	1–5	11	N° of PCR positive transgenic shoots/weight (g) calli used for transformation	Wang et al., 2017a
Biolistic	Badila	*npt*II	53	1–3	0.58	13 southern blotting positive lines from 2251 bombarded calli	Yao et al., 2017
*Agrobacterium*	ROC22	PMI	33	0–7	7.3	32 transgenic lines PCR positive/4.3 g fresh weight of callus	Wang et al., 2017b
Biolistic	SPF-234 and NSG-311	*hpt*II	4	1	29%	N° of PCR and southern blot positive regenerates plant	Aslam et al., 2018
Biolistic	*Saccharum spp.* hybrids	*bar*	55	1–8	39%	N° of independent transgenic lines expressing the gene of interest/n° of bombardments	Ramasamy et al., 2018
*Agrobacterium*	Co 86032	*hpt*II	6	1–3	35.8%	N° of GUS positive plants/total n° of infected callus	Sathish et al., 2020

Interestingly, there are only two major studies published where a direct efficiency comparison between *A. tumefaciens* and biolistic transformation in sugarcane was performed. Somewhat surprisingly, in the first study the efficiency differences reported were in favor of the biolistic method (Jackson et al., 2013), while the second study showed no significant difference between them (Wu et al., 2015). Taken together these results suggest that there is very little or no difference between the two methods and both are equally likely to generate transformants suitable for research studies or commercial release.

For breeding purposes it is recommendable to obtain transgenic events with a low copy number, which is also an important advantage when performing genetic studies for a commercial release. To correctly establish the number of transgene insertions in the plant genome, experiments of Southern blot hybridization are essential, although impractical for screening numerous potential events obtained under a scheme of high scale transgenic plant production. Therefore, a relatively rapid preliminary screen to find low copy number events based on quantitative real-time polymerase chain reaction (qRT-PCR) has successfully been employed. Jackson et al. (2013) determined a copy number index score from qRT-PCR of 196 transgenic plants, which were correlated (R2 = 0.65) to the number of hybridizing bands in Southern blot experiments for 19 of the tested lines. In another experiment Gao et al. (2016) established a standard curve from serially diluted plasmid DNA to estimate the copy number of the *cry1Ac* gene in transgenic sugarcane. Copy numbers estimated from qRT-PCR experiments of 14 transgenic lines ranged from 1 to 148 and from these six different lines were selected to determine copy number by Southern blot. Results showed that copy numbers estimated by qRT-PCR were higher than those estimated by Southern blot analysis, although the overall trend of copy number estimation by the two methods were consistent. The usefulness of this approach for large-scale screening was demonstrated by Cristofoletti et al. (2018), who estimated copy numbers in transgenic sugarcane expressing two Bt genes in 236 events, using 5–7 plants with previously established insertion copy number as controls.

In sugarcane, numerous transformation events obtained with *A. tumefaciens* have been shown to be single transgene events indicating a high percentage of single gene incorporation by this method. Nevertheless, most reports on *A. tumefaciens*-mediated transformation only analyzed a limited number of plants (less than 7; Table 1) by Southern blot. There are; however, a few studies conducted where a high number of transgenic events have been analyzed by Southern blot, both for *A*. *tumefaciens* and biolistic transformation. When Mayavan et al. (2015) studied transgene copy number in 146 *A*. *tumefaciens*-transformated events, they found that 25% carried a single copy and 75% 2–3 copies. In a similar study Wang et al. (2017b) analyzed 33 transgenic events derived from *A. tumefaciens*-mediated transformation and reported 15% of single-copy events and 75% of plants with 2–7 copies. In addition, when 48 transgenic sugarcane plants derived from particle bombardment were analyzed, 19% were defined as single copy events and 58% with less than four insertions (Taparia et al., 2012b). More recently, Ramasamy et al. (2018) evaluated 55 transgenic plants generated by biolistic transformation where 30% were single copy events and 70% with 2–7 copies. Taken together these studies suggest that there are very little differences between the two methods, and single insertion events are frequently encountered by both techniques.

Another important determinant of the overall success of generating a transgenic event and the introduced character is the level of expression of the transgene. This depends on numerous factors, where some variables can be controlled by the researcher like selection of promoters (Liu et al., 2003; Petrasovits et al., 2012), nucleotide optimization of genes and codon usage (Jackson et al., 2014); while other key variables that cannot be manipulated include insertion site in the plant genome and gene silencing. In sugarcane several studies have demonstrated that there is no or very little correlation between the number of inserted copies of a transgene and its expression levels (Jackson et al., 2013; Joyce et al., 2014; Wu et al., 2015), suggesting that the major factor determining expression is correlated to the insertion site. One promising method to reduce the position effects on expression levels of transgenes in plants is the introduction of insulator sequences, which establish genomic barriers to adjacent DNA sequences and thereby protect genes from the influence of neighboring heterochromatin regions (West et al., 2002). In a relatively recent study a significant increase, more than twofold, in *npt*II expression was observed in sugarcane plants transformed using an expression cassettes flanked by the two insulator sequences, EXOB and TBS (Zhao et al., 2019). In the same study, intent to develop a defined area for transgene introduction (gene stacking) in the sugarcane genome was developed by the introduction of an Ubiquitin promoter adjacent to a *lox76* site. The idea being to support site-specific integration of a promoter-less selectable marker construct with additional transgenes into the *lox76* site to ensure high and stable gene expression levels and facilitate commercial release by a defined transgene integration site (Zhao et al., 2019).

Regarding gene silencing, analysis of transgenic sugarcane has shown a highly efficient and rapidly imposed silencing of diverse transgene constructs (Ingelbrecht et al., 1999; Wei et al., 2003; Mudge et al., 2009; Birch et al., 2010). To better understand this effect Jackson et al. (2014) studied different aspects of gene silencing and concluded that elimination of sequences implicated in RNA instability concurrently with rare codons and undesired structural features such as repeat sequences significantly reduced silencing in transformed sugarcane. Another strategy to significantly decrease gene silencing in transgenic event includes the incorporation of post-transcriptional gene silencing (PTGS) suppressors (Gao et al., 2013).

In summary, evidence has demonstrated that commercially acceptable transgenic sugarcane plants can be generated by using either *A. tumefaciens* (in an amenable genotype) or biolistic-mediated transformation methods. Both methods require the generation of a high number of transgenic events to identify those with low copy number expressing transgene at required levels for specific traits, while maintaining the rest of the genome practically unaltered.

## Resistance to Biotic Stresses

Sugarcane production, like most other crops, is strongly influenced by the impacts of biotic and abiotic stresses, which are the main reason for the large differences observed between the average and the maximum potential yield in most, if not all, crops (Babu et al., 2021). In sugarcane production, diseases, pests and undergrowth are considered the most important biotic stresses affecting yields.

### Resistance to Diseases

In general, in modern sugarcane production systems, diseases are predominantly controlled by an integrated approach involving the combination of disease-free planting material, disease-resistant cultivars, applicable farm management practices, and strict quarantine measures (Babu et al., 2021). A broad disease resistance is an important part of sugarcane breeding as diseases occurs annually in all sugarcane production areas causing significant losses in production. Actually, more than 100 pathogens (including bacteria, fungi, viruses, phytoplasmas, and nematodes) have been recognized as causal agents of diseases in sugarcane (Rott, 2000; Govindaraju et al., 2019) and therefore, screening and breeding sugarcane for disease resistance is a very important process in all breeding programs (Rahman et al., 2019). Nevertheless, although sugarcane breeders try to select for resistant genotypes, it is an almost overwhelming challenge to introduce resistance against all pathogens at the same time through conventional breeding (Cursi et al., 2021a), and as a consequence many commercial varieties are susceptible to more than one pathogen. It is also important to clarify that many high-yielding clones obtained by selection in breeding programs do not reach a commercial release due to their high susceptibility to various pathogens (Babu et al., 2021). This fact has led to considerable research efforts to generate knowledge and develop molecular breeding strategies to provide durable disease resistance in combination with superior agronomic performance in commercial clones (Tiwari et al., 2010; Table 2).

**TABLE 2:** List of transgenic sugarcane engineered for disease resistance.

Type of explant	Promoter	Method of transformation	Transgene	Target disease	Variety	References
Calli	Emu and Ubi	Particle bombardment	*CP*	SCMV	Q95, Q153 and Q155	Joyce et al., 1998
Calli	Maize Ubi-1	Particle bombardment	*CP*	SrMV	CP65-357 and CP72-1210	Ingelbrecht et al., 1999
Calli	Ubi	Particle bombardment	Albicidin detoxifying	Leaf scald	Q63 and Q87	Zhang et al., 1999
Calli		*Agrobacterium*	Glucanase and a chitinase	Brown rust	Ja60-5 and B4362	Enriquez et al., 2000
Calli	Ubi	Particle bombardment	Segment 9 of ORF 1	FDV	Q124	McQualter et al., 2004
Calli	Ubi	Particle bombardment	*CP*	SCMV	CP 84-1198 and CP 80-1827	Gilbert et al., 2005
Calli	Ubi	Particle bombardment	*CP*	SCYLV	CP 92-1666	Gilbert et al., 2009
Cell cultures	Ubi	Particle bombardment	*CP*	SCYLV	H62-4671	Zhu et al., 2011
Leaf	35S	*Agrobacterium*	*CP*	SrMV	ROC22	Guo et al., 2015
axillary bud	35S	*Agrobacterium*	β-1,3-glucanase	Red rot	CoJ 83	Nayyar et al., 2017
Shoots	35S	*Agrobacterium*	*CP*	SCMV	Bululawang	Apriasti et al., 2018
Calli	Ubi	Particle bombardment	*CP*	SCMV	SPF-234 and NSG-311	Aslam et al., 2018
Calli	Ubi	Particle bombardment	Chitinase class-II	Red rot	S2006SP-93	Tariq et al., 2018

The incidence of viral diseases is steadily increasing in sugarcane and breeding for virus disease resistance is therefore an important research topic (Rahman et al., 2019), which have included various strategies of genetic engineering to generate resistant varieties. Earlier transformation strategies were primarily based on virus capsid protein (CP) and movement protein-mediated protection, where a transgene derived homolog of a viral protein was expressed in plants, which interferes with or prevents various stages of the viral life cycle, resulting in an attenuated disease symptom or resistance (Ingelbrecht et al., 1999). However, more recent works have predominantly been based on producing viral resistant plants by using RNA interference (RNAi) technology. RNAi is an evolutionarily conserved process of sequence-specific PTGS in both animals and plants, initiated by double-stranded RNA (dsRNA) that is homologous in sequence to the silenced gene. The dsRNA or hairpin RNA (hpRNA) are processed into 21–24 nucleotide (nt) small interfering RNA (siRNA) duplex by Dicer or Dicer-like (DCL) protein and into 21–22 nt siRNA by ribonuclease III cleavage from longer dsRNAs, which further mediate sequence-specific mRNA degradation (Viswanathan et al., 2014).

The two major virus diseases in sugarcane are Mosaic, caused by Sugarcane Mosaic Virus (SCMV) and Sorghum Mosaic Virus (SrMV) (Yang and Mirkov, 1997; Perera et al., 2009) and yellow leaf syndrome, caused by Sugarcane Yellow Leaf Virus (SCYLV) (Bertani et al., 2014). Both diseases have been reported in almost all sugarcane producing areas worldwide (Grisham, 2000; Ahmad et al., 2007) and considering the economic impact and wide-spread of these viruses, many genetic transformation strategies have been implemented to obtain resistant plants. In one of the first attempts to generate virus resistance, Joyce et al. (1998) transformed sugarcane plants with the SCMV *CP* gene by microprojectile bombardment and ten of the transgenic lines demonstrated resistance when challenged with the virus. In another study, Ingelbrecht et al. (1999) developed transgenic sugarcane plants derived from an untranslatable form of the SrMV strain SCH *CP* gene. Transgenic events, when challenged with the virus, showed a wide range of responses from fully susceptible to completely resistant phenotypes. In a similar work, the gene encoding the CP from SCMV was amplified by RT-PCR from symptomatic sugarcane leaves and used to generate transgenic sugarcane. Full (927 bases pairs, bp) and N-terminally truncated (702 bp) sequences were used to generate constructs and introduce them into sugarcane by *Agrobacterium*-mediated transformation. Artificial infection by the virus showed that the full sequence generated a better protection against the virus compared to the truncated one. Interestingly, the resistance was passed on to the second generation of transgenic sugarcane with higher levels of resistance demonstrated for lines transformed with the complete *CP* sequence. These results suggested that the complete sequence of the *CP* gene was required to disrupt viral assembly and packaging, thereby generating resistance to SCMV infection (Apriasti et al., 2018).

Zhu et al. (2011) produced SCYLV-resistant transgenic sugarcane from a susceptible through biolistic bombardment of cell cultures with an untranslatable *CP* gene. The resistance level, in some of the transgenic events as measured by virus titer and disease symptom development, was similar to that of a completely resistant cultivar.

In another study, transgenic lines with significantly enhanced resistance to Fiji disease virus (FDV) were obtained through microprojectile-mediated transformation using a transgene encoding a translatable version of FDV segment 9 of ORF 1 of the virus genome. Resistance of transgenic lines was tested in glasshouse trials and only one transformed line showed significant enhanced resistance to Fiji disease compared to the parental genotype. However, the molecular phenotypes of transgenic plants were not entirely consistent with a resistance mechanism solely based on PTGS (McQualter et al., 2004).

A direct RNA silencing strategy was used for the production of anti-SrMV sugarcane plants by the generation of RNAi to suppress *CP* gene expression (Guo et al., 2015). Based on multiple sequence alignments of the conserved region of the *CP* gene of different strains and isolates of SrMV, this gene was selected as the RNAi target and the interference sequence was obtained through PCR amplification. The RNAi vector with an expression cassette encoding a hairpin interference sequence was transferred to sugarcane *via Agrobacterium*-mediated transformation. After artificial inoculation challenge, anti-SrMV positive transgenic lines were successfully obtained with a resistance rate of up to 87.5%. With a similar strategy, Aslam et al. (2018) generated transgenic sugarcane plants expressing short hairpin RNAs (shRNA) targeting the *CP* gene of SCMV. After mechanical inoculation of transgenic and non-transgenic sugarcane events with SCMV, a variable degree of resistance was found, where some events showed resistance close to immunity against SCMV infection. These results demonstrated that small RNAs, processed from integrated pre-shRNA fragments and produced by transgenic sugarcane plants, can induce RNAi and silencing upon virus inoculation.

There is, to the best of our knowledge, only one study published on genetic engineering for control of a bacterial disease in sugarcane, which is based on plants expressing an albicidin detoxifying gene (*albD*), cloned from a bacterium that provides biocontrol against leaf scald disease, caused by *Xanthomonas albilineans* (Zhang et al., 1999). Transgenic plants accumulating AlbD did not develop chlorotic disease symptoms in inoculated leaves, whereas all non-transformed control plants developed severe disease symptoms. In addition, transgenic lines with high AlbD activity in young stems were also protected against systemic multiplication of the pathogen.

In one of the first studies to control fungal diseases through genetic engineering in sugarcane, a glucanase and a chitinase genes were expressed to confer resistance against *Puccinia melanocephala*, the causal agent of brown rust, but no results on resistance to infections by the fungus have been published (Enriquez et al., 2000). In a similar approach, Nayyar et al. (2017) reported the development of red rot resistant transgenic sugarcane through expression of a β-1,3-glucanase gene from *Trichoderma* spp. Bioassays of transgenic plants with virulent *Colletotrichum falcatum* strains, the causal agent of red rot, demonstrated different levels of resistance in transgenic lines. It must be pointed out that electron micrographs of sucrose storing stalk parenchyma cells from these plants, the main target of attacks of this fungus, displayed characteristic sucrose-filled cells inhibiting *C. falcatum* hyphae growth. Transgene expression was up-regulated after infection, and the transgene was successfully transmitted to a second clonal generation raised from resistant transgenic plants. More recently, Tariq et al. (2018) evaluated sugarcane transgenic lines expressing a barley chitinase class-II gene, for protection against *C. falcatum* infection. Crude protein extracts from transgenic sugarcane plants inhibited the mycelial growth of *C. falcatum* in a quantitative *in vitro* assay and two transgenic lines exhibited strong protection against inoculated *C. falcatum* in an *in vitro* bioassay. The mRNA expression of the transgene in the *C. falcatum*-inoculated transgenic sugarcane lines increased gradually compared to control parental plants.

There are only a few published field studies on disease resistant transgenic sugarcane, but Gilbert et al. (2005) evaluated variability in agronomic characteristics and field disease resistance of two sugarcane cultivars, transformed for resistance to SCMV strain E and found a large variability in both yield characteristics and disease resistance. In another trial, Gilbert et al. (2009) performed a 3-year study to evaluate both agronomic performance and virus resistance of transgenic lines transformed for SCYLV resistance by antisense expression of a part of the virus *CP* gene. Sugarcane yields of parental genotype were superior to transgenic lines, but SCYLV infection rates in transgenic lines were only 0–5%, compared to 98% in the parental variety. Differences in yield could be explained by great genetic variation caused by somaclonal variation during the *in vitro* regeneration process of transgenic lines.

More recently, a 2-year field study was performed to compare transgenic sugarcane lines expressing the SCMV-CP gene to improve virus resistance. Agronomic performance, resistance to SCMV infection, and transgene stability were evaluated and compared with the wild-type parental clone Badila at four experimental locations in China across two successive growing seasons (plant cane and first ratoon age). All evaluated transgenic lines produced significantly greater amount of cane and sucrose per hectare as well as a lower SCMV disease incidence, when compared to the parental variety at both plant ages (Yao et al., 2017).

Results from several studies revealed that, although the level of genomic changes in transgenic events was low, most transgenic events had undergone minor but clear morphological, physiological, and phytopathological variations. For that reason, the extent of somaclonal variation should be adequately determined in any transgenic populations in order to allow an appropriate management and evaluation of field trials (Noguera et al., 2015; Nerkar et al., 2018).

### Resistance to Pests

Another significant limiting factor influencing sugarcane production is the damage brought about by different stem borers from the Order of Lepidoptera (Weng et al., 2011). The major Lepidopteran insect pests of sugarcane are: the stem borer (*Diatraea saccharalis*) in South America, Central America, the Caribbean and southern parts of United States (De Souza Rossato et al., 2011); the root borer (*Emmalocera depressella*) in India and Pakistan; the sugarcane top borer (*Chilo terrenellus*) in Bangladesh, Thailand and Australia (Goebel and Way, 2003); the pink borer (*Sesamia inferens*) in Cambodia, China, Hong Kong and India; the Mexican rice borer (*Eoreuma loftini*) and pink stem borer (*Sesamia cretica*) in the Mediterranean basin, the Middle East and Arabia, Pakistan, India, northern Africa, part of Kenya and Cameroon. All these borers can cause significant losses in both biomass and sugar content and have an important economic impact (Iqbal et al., 2021).

Advances in genetic transformation technology have led to rapid progress in the genetic engineering of plants for protection against pests by transferring genes derived from plants, pests, and bacteria (Gill et al., 2006; Romeis et al., 2006; Schneider et al., 2017; Gosal and Wani, 2018; Table 3). Several insecticidal proteins of plant origin such as lectins and protease inhibitors (PIs) can retard insect growth, development and reproduction when ingested by insects at high doses. Some genes like *avac* (*Amaranthus viridis* L. agglutinin), *skti* (soybean Kunitz trypsin inhibitor), *sbbi* (Bowman–Birk inhibitor), and *gna* (*Galanthus nivalis* agglutinin) have all been used in genetic transformation programs aiming at developing insect resistance in sugarcane (Sétamou et al., 2002; Falco and Silva-Filho, 2003; Deng et al., 2008).

**TABLE 3:** List of transgenic sugarcane engineered for pest resistance.

Type of explant	Promoter	Method of transformation	Candidate gene	Target Pest	Variety	References
Calli	CaMV35S	Electroporation	*cry1Ab*	*D. saccharalis*	Ja 60-5	Arencibia et al., 1997
Not reported	Maize ubi-1	Particle bombardment	*Gna*	*Antitrogus consanguineous*		Allsopp et al., 2000; Nutt et al., 2001
Calli	Maize PEPC	Particle bombardment	*cry1Ab*	*D. saccharalis*	SP80-1842	Braga et al., 2001, 2003
Not reported	Maize Ubi-1	Paint-sprayer delivery	Snowdrop lectin	*Eoreuma loftini; D. saccharalis*	CP65-357	Sétamou et al., 2002
Calli	Maize Ubi-1	Particle bombardment	Soybean Kunitz trypsin inhibitor (*skti*) Soybean Bowman-Birk inhibitor (*sbbi*)	*D. saccharalis*	SP80-1842 and SP80-3280	Falco and Silva-Filho, 2003
Calli	Maize Ubi-1	Particle bombardment	Synthetic-*cry1Ac*	*P. venosatus*	YT79-177 and ROC16	Weng et al., 2006
Calli	RSs-1 Maize Ubi-1	*Agrobacterium*	*Gna*	*Ceratovacuna lanigera*	FN81–745 and Badila	Zhangsun et al., 2007
Calli	Ubi-1	*Agrobacterium*	Fusion *Amaranthus viridis* agglutinin and *skti* genes	*D. saccharalis*	ROC25	Deng et al., 2008
Calli	Ubi	Particle bombardment	*cry1Ac*	*D. saccharalis*	Gui94-119	Xu et al., 2008
Calli	Maize ubi-1	Plasmid transformation	*HIS Cane CPI – 1*	*Sphenophorus levis*	SP80-185	Ribeiro et al., 2008
Leaf roll	CaMV35S	*Agrobacterium*	*cry1Aa3*	*C. infuscatellus, C. sacchariphagus* and *S. excerptalis*	CoC671	Kalunke et al., 2009
Calli	Maize Ubi-1	Particle bombardment *Agrobacterium*	*cry1Ab*	*C. infuscatellus*	Co 86032 and CoJ 64	Arvinth et al., 2010
Calli	Maize Ubi-1	Particle bombardment	*Aprotinin*	*S. excerptalis*	CoC 92061 and Co 86032	Christy et al., 2009
Calli	Maize Ubi-1	Particle bombardment	Modified-*cry1Ac*	*P. venosatus*	YT79-177 and ROC16	Weng et al., 2011
Calli	CaMV35S	Particle bombardment	*cry1Ac*	*D. saccharalis*	FN15	Gao et al., 2016
Calli	CaMV35S	*Agrobacterium*	*cry1Ab*	*D. saccharalis*	LK 92-11	Islam et al., 2016
Calli	Maize ubi-1	Particle bombardment	*CaneCPI-1*	*Sphenophorus levis*	SP80-185	Schneider et al., 2017
Calli	Ubi-1	*Agrobacterium*	*cry1Ab*	*D. saccharalis*	ROC22	Wang et al., 2017b
calli	CaMV35S	Particle bombardment	*cry1Ac*	*D. saccharalis*	FN15 and ROC22	Zhou et al., 2018
Calli	CaMV35S and FMV	*Agrobacterium*	*cry1Ab* and *cry2Ab*	*D. saccharalis*	SP 803280	Cristofoletti et al., 2018
Calli	ST-LSI	Particle bombardment	*cry2A*	*C. sacchariphagus, S. nivella, C. infuscatellus, A. schistaceana and S. inferens*	ROC22	Gao et al., 2018
Calli	PEPC	*Agrobacterium*	*cry1Ab*	*D. saccharalis*	Event CTC175-A	Cheavegatti-gianotto et al., 2018
Calli	Maize ubi-1	*Agrobacterium*	*cry1ac*	*D. saccharalis*	Event CTC91087-6	Gianotto et al., 2019
Calli	RUBISCO	*Agrobacterium*	*cryIAb-CryIAc*	*Scripophaga excerptalis*	Bululawang	Koerniati et al., 2020
Young leaf	CaMV 35S	*Agrobacterium*	*cry1Ac*	*Sesamia cretica*	GT54-9(C9)	Dessoky et al., 2020
Calli	Maize Ubi-1	*Agrobacterium*	*Vip3A*	*Chilo infuscatellus*	CPF-246	Riaz et al., 2020

*Table adapted from Iqbal et al. (2021).*

Transgenic sugarcane plants engineered to express either the potato proteinase inhibitor II or the snowdrop lectin gene, showed increased antibiosis to larvae of the canegrub *Antitrogus consanguineus* in glasshouse trials. Canegrubs feeding on the transgenic line, transformed with the potato gene, gained 4.2% of the weight gain of canegrubs fed on untransformed control plants. Similarly, larvae feeding on the roots of transgenic line, transformed with the snowdrop gene, gained only 20.6% of the weight gain of grubs feeding on the non-transgenic control plants (Allsopp et al., 2000). Similarly, larvae from the Greyback Cane Beetle (*Dermolepida albohirtum)* fed on transgenic plants expressing the snowdrop lectin and the proteinase inhibitor, decreased in weight compared to larvae fed on non-transformed plants (Nutt et al., 2001). Likewise, growth of larvae of *D. saccharalis*, fed on transgenic sugarcane transformed with *skti* and *sbbi* from soybean, was considerably restricted compared to larvae from control plants (Falco and Silva-Filho, 2003). *In vivo* bioassay studies of transgenic sugarcane transformed with a synthetic bovine pancreatic trypsin inhibitor (aprotinin) gene for protection against the top borer (*Scirpophaga excerptalis*), indicated that larvae fed on transgenic plants exhibited extensive decrease in larval weight up to 99.8% (Christy et al., 2009). Schneider et al. (2017) produced transgenic sugarcane lines overexpressing sugarcane cysteine peptidase inhibitor 1 (*CaneCPI-1)* and evaluated their potential resistance through feeding assays with larvae of the sugarcane billbug (*Sphenophorus levis*). Significantly less damage by larval attack was caused on transgenic plants compared to non-transgenic parental variety plants.

The trypsin inhibitory activity of PIs from *Erianthus arundinaceus*, a wild relative of sugarcane, was evaluated and shown to differ significantly among different plant parts. The highest inhibition activity was found in meristematic tissue and PIs isolated from the apical meristem effectively inhibited midgut proteinases of both the early shoot borer (*Chilo infuscatellus*) and internode borer (*Chilo sacchariphagus indicus*) (Punithavalli and Jebamalaimary, 2019; Punithavalli, 2021). These studies show the possibility of identifying new and efficient PIs to produce sugarcane plants resistant to stem borers.

Nonetheless, the most important insecticidal proteins are produced by the Gram-positive spore forming bacteria *Bacillus thuringiensis* (Bt), which produces proteins both during its vegetative phase (Vip) and sporulation phase (Cry) which are very toxic to a wide range of insects (Chakroun et al., 2016). Insecticidal proteins from Bt have been widely used to produce transgenic plants to control many different insect pests because as they are highly efficient against their targets but not toxic to vertebrates or other insects (Baranek et al., 2017). Although Cry and Vip toxins have the same general mode of action, they have no structural homology and bind to different sites in the larval midgut, which suggests that strong cross-resistance between them is highly unlikely (Carrière et al., 2016; Chakroun et al., 2016).

Crops genetically engineered to produce insecticidal proteins from *Bt* have revolutionized control of some major lepidopteran and coleopteran pests in important crops such as corn and cotton (Tabashnik et al., 2020), but even more significantly the development of Bt crops has been extremely important in dramatically reducing the use of harmful chemical insecticides in agriculture (Qamar et al., 2015).

The success of Bt crops in agriculture production generated an early interest to use Bt toxins to control pests in sugarcane, and especially stem borers. In 1997, the development of the first transgenic sugarcane resistant to *D. saccharalis*, carrying the *cry1Ab* gene, was published (Arencibia et al., 1997) and after this first successful event, several works have been conducted in which different *Bt* genes, such as *cry1Ab*, *cry1Aa3*, *cry1Ac*, *s-cry1Ac*, *m-cry1Ac, cry2A* and *vip3A*, have been introduced in different sugarcane genotypes and shown to improve resistance to different Lepidopteran pests (see Table 3). In initial studies the effectiveness of transgenic events were demonstrated in laboratory tests but in the last decade several field tests of transgenic sugarcane expressing Bt toxins have been conducted demonstrating their effectiveness against various pests.

In an attempt to increase the insecticidal activity of a truncated *cry1Ac* gene, which encoded the active region of Cry1Ac insecticidal δ-endotoxin, the GC-content was increased from the original 37.4–47.5% following the sugarcane codon usage pattern (s-Cry1Ac). Four transgenic lines accumulating detectable amount of the truncated s-Cry1Ac protein were assayed for stem borer resistance in leaf tissue feeding trials and greenhouse whole plant assays. Results showed that, while untransformed control lines were severely damaged, all transgenic sugarcane lines were highly resistant to stem borer attack, which resulted in complete mortality of inoculated larvae within 1 week after inoculation (Weng et al., 2006). In a subsequent study by the same research group, the GC content of the truncated insecticidal gene (*m-cry1Ac*) was further increased to 54.8%. Transgenic plants expressing *m-cry1Ac* produced five times more Cry1Ac protein than plants transformed with the previous synthetic s-Cry1Ac. Interestingly, transgenic plants accumulating high levels of m-Cry1Ac were completely resistant against stem borer in both field and greenhouse trials, whereas transgenic *s-cry1Ac* sugarcane plants demonstrated an intermediate resistance (Weng et al., 2011).

Using the same gene, *cry1Ac*, Gao et al. (2016) demonstrated that transgenic plants accumulating high levels of the complete Cry1Ac protein showed resistance to stem borer attack both in greenhouse bioassay experiments and in repeated field trials. The phenotypic traits of many transgenic lines with a high Cry1Ac accumulation and stem borer resistance were found to be very similar to non-transformed parental lines. In another study, Gao et al. (2018) transformed sugarcane with the *cry2A* gene by particle bombardment. Transgenic plants expressing *cry2A* showed a significantly increased protection against *D. saccharalis* and comparable sugar yield to non-transformed parental genotype.

In a more in depth study, Cristofoletti et al. (2018) expressed two Bt proteins with different modes of action (Cry1Ab and Cry2Ab) in the same plant of a commercial variety of sugarcane, demonstrating not only the efficacy against sugarcane borer in the field but also the possibility to generate transgenic lines with the same agronomic characters as the parental elite variety. Furthermore, this study presents evidence that a strategy including two Bt toxins with different mode of action and high accumulation in the plant tissue is a viable approach to avoid insect resistance in a sufficient number of insect generation (100) if combined with a well-thought refuge strategy.

In a recent work, Riaz et al. (2020) developed transgenic sugarcane lines expressing the Vip3A toxin against *C. infuscatellus*. The transgenic lines showed increased resistance to the stem borer (up to 100% mortality) compared to non-transformed control sugarcane line. Their findings also suggested that the *vip3A* gene could be employed in gene pyramiding with other Bt toxins for a more effective and prolonged resistance as suggested by Cristofoletti et al. (2018).

As a consequence of the massive adoption of Bt crops in many countries, cases of resistance in target pest populations to certain Bt events have been detected (Tabashnik et al., 2013; Trumper, 2014). For this reason, different strategies have been developed to delay resistant evolution in susceptible insects, that includes the delivery of high doses of protein, crop genetically engineered to produce two or more distinct toxins that kill the same pest (pyramided transgenic crop) and the use of a refuge (Cristofoletti et al., 2018). Considering that the sugarcane borer is capable of completing up to five generations per year in South America (Guevara and Wiendl, 1980; de Melo and Parra, 1988; Salvatore et al., 2009), it is recommended that the development of multi-toxin transgenic sugarcane will need to control at least 100 generations, to provide a sufficient commercial life span of a variety (Cristofoletti et al., 2018). Pyramided Bt genes have been rapidly adopted in other crops and are expected to be even more frequent in the future, including sugarcane.

The efficient control of stem borers by employing Bt toxins in sugarcane, demonstrated in the studies described above, resulted in the first commercial release of a transgenic sugarcane variety in 2017, when event CTB141175/01A expressing the *cry1ab* gene was officially approved for production in Brazil. More recently two other sugarcane events, CTC91087-6 and CTC93209-4, were released in the same country, both expressing the *cry1Ac* gene (ISAAA, 2021).

### Resistance to Herbicides

The use of herbicides has revolutionized weed control in many crop production systems, but as herbicide resistance in many weeds increases with prolonged application, new approaches are needed to maintain the efficiency of chemical weed control including the discovery of new herbicide target sites in plants and the discovery and synthesis of new, more potent herbicidal molecules. As these approaches are expensive, time consuming and eventually lead to increased chemical loads in the environment, an alternative strategy was to apply biotechnological techniques to develop crops with resistance to broad spectrum herbicides to ensure a safer and more efficient use in a wide range of crops (Mulwa and Mwanza, 2006). For this reason, the development of herbicide-resistant crops was one of the first commercial applications of plant genetic engineering (Leibbrandt and Snyman, 2003) and in the last decade more than 80% of the land planted with genetically modified crops was planted with glyphosate- or glufosinate-resistant species (Wang et al., 2017b). Sugarcane is mainly planted on tropical and subtropical dry farmland with high precipitation, which normally implies a significant weed infestation leading to competition for nutrients, water and sunlight for survival, and as a consequence important reduction in crop yields takes place (Nasir et al., 2013). Furthermore, the absence of herbicide-resistant genes in the genetic pool of wild relatives of sugarcane makes traditional breeding for this trait almost impossible and leaves genetic engineering as an amenable approach to produce herbicide-resistant varieties in sugarcane.

The first attempt to introduce herbicide resistance in sugarcane was made by Chowdhury and Vasil (1992) when they transformed suspension culture cells by microprojectile bombardment and by electroporation of protoplasts with a plasmid containing the bacterial gene *bar* (phosphinotrycin acetyltransferase), a selectable marker which confers resistance to the herbicide Basta^®^ (glufosinate ammonium). Colonies resistant to Basta from both transformation systems were recovered and stable integration of *bar* gene was confirmed by Southern blot analysis. However, no whole plants were regenerated since cell lines were old and non-morphogenic. Glufosinate ammonium is a non-selective pro herbicide that is converted by plants into the phytotoxin, phosphinothricin (PPT). The herbicide acts by inhibiting the essential ammonia assimilation enzyme, glutamine synthetase (Mulwa and Mwanza, 2006). The first transgenic sugarcane expressing the *bar* gene was produced by Gallo-Meagher and Irvine (1996) by biolistic transformation of embryogenic calli. When field performance of glufosinate ammonium-resistant transformants was assessed, stable transgene expression was observed over several ratoons with repeated herbicide Ignite application, either to leaf segments or to the entire plant with lethal dosages for non-transformed control plants. Interestingly, when transgenic events were propagated through meristem propagation culture, resistance was conserved and these results demonstrated that *bar* could be used as an effective selectable marker in sugarcane transformation. Likewise, Snyman et al. (1998) transformed embryogenic callus of the same variety, NCO310, by microprojectile bombardment using a vector containing a synthetic *pat* gene, which also confers resistance to glufosinate ammonium (Buster^®^). Regenerated transgenic lines expressing *pat* was field tested and results demonstrated that the herbicide-resistant gene was stably expressed during three rounds of vegetative propagation. Morphological and agronomic characters such as stalk height, diameter, population, fiber, disease resistance and yield, measured in the first ratoon, were not significantly different between transgenic lines and non-transformed plants (Leibbrandt and Snyman, 2003). In addition, Enríquez-Obregón et al. (1998) genetically transformed the variety Ja69-15 by *Agrobacterium-*mediated transformation introducing the *bar* gene and obtained Basta-resistant calli. These reports and others of transgenic sugarcane events resistant to glufosinate ammonium are summarized in Table 4.

**TABLE 4:** Reports of herbicide resistant sugarcane transgenic events.

Herbicide resistance	Type of explant	Method of transformation	Promoter	Gene	Variety	References
Glufosinate ammonium (Basta, Buster, Ignite)	Suspension cells	Particle bombardment	Adh1	*Bar*	CP72-1210	Chowdhury and Vasil, 1992
	Protoplast	Electroporation				
	Embryogenic calli	Particle bombardment	Ubi-1	*Bar*	NCo 310	Gallo-Meagher and Irvine, 1996
	Embryogenic calli	Particle bombardment		*Pat*	NCo 310	Snyman et al., 1998
	Meristematic explants	*Agrobacterium*	Rice ubiquitin	*Bar*	Ja60-15	Enríquez-Obregón et al., 1998
	Embryogenic calli	Particle bombardment	Ubi-1	*Bar*	SP80–180	Falco et al., 2000
	Axillary bud explants	*Agrobacterium* strains LBA4404 and EHA105	CaMV 35S	Bar	Co92061 and Co671	Manickavasagam et al., 2004
As effective as Buster	Somatic embryos	Particle bombardment	CaMV 35S		N12 and N19	Snyman et al., 2001
Glyphosate (Round up)	Embryogenic calli	Particle bombardment	CaMV 35S	Glyphosat tolerant	CPF-234, CPF-213, HSF-240 and CPF-246	Nasir et al., 2013
	Embryogenic calli	Particle bombardment	Rice Actin	*Epsps*	RA87-3	Noguera et al., 2015
	Embryogenic calli	*Agrobacterium*	Ubi-1	*Epsps (Cry1Ab)*	ROC22	Wang et al., 2017b
	Embryogenic calli	Particle bombardment	Rice Actin	*Epsps*	TUC 03-12	Racedo et al., 2019
	Leaf roll disk					
ALS-inhibiting herbicides	Embryogenic calli	Particle bombardment	CRISPR/Cas9	*ALS*		Tufan Oz et al., 2021

It must be pointed out that weed control treatment is dependent on the cost of the herbicide to which resistance has been engineered. In that respect, the cost of Basta^®^/Buster^®^ herbicide is relatively high and therefore a gene conferring resistance to herbicide (HR) a fivefold cheaper was introduced into commercial sugarcane cultivars N12 and N19. Gene delivery by microprojectile bombardment was accomplished using five different plasmid constructs, each containing the same herbicide resistance gene (gene not revealed) regulated by different promoters and the antibiotic selectable marker gene *nptII*. Plantlets were regenerated *via* either direct or indirect somatic embryogenesis and putatively transformed plants were subjected to herbicide spraying in glasshouse where 52% of the transgenic plants survived a sub-lethal dose, which severely damaged control plants (Snyman et al., 2001).

Without any doubt the development of glyphosate (Roundup^TM^) resistant soybean and maize in the late nineties transformed weed control and crop production worldwide. In 2019 more than 190 million hectares were planted with transgenic crops and out of those 88% were planted with glyphosate-resistance plants (ISAAA, 2019). Glyphosate is a very effective broad spectrum herbicide that inhibits the enzyme 5-enolpyruvylshikimate phosphate (EPSP) synthase necessary in the biosynthetic pathway of the aromatic amino acids, tryptophan, tyrosine, and phenylalanine, which are essential for protein synthesis and also as precursors for hormones, lignins, and other protective compounds such as flavanoids and alkaloids (Amrhein et al., 1980). EPSP synthase uses phosphoenol pyruvate (PEP) and shikimate-3-phosphate as substrates to make EPSP but glyphosate competitively interferes with the binding of PEP to the active site of EPSP synthase, hence blocking the pathway (Anderson et al., 1988).

Although the success of glyphosate resistance has been demonstrated in various crops since the late nineties it is not until the last decade that efforts to produce glyphosate-resistant sugarcane have been reported. The first published study included the genetic transformation of four sugarcane varieties through bombardment of embryonic calli with an unnamed glyphosate tolerant (GT) gene Nasir et al. (2013). Almost all transgenic plants (88%) survived a first application of glyphosate (900 mL in 80 L of water), while all non-transformed plants died within a week after treatment. In a second application with a higher glyphosate concentration, only plants with high accumulation of the GT gene encoded protein survived. In addition, all weeds growing alongside transgenic sugarcane plants turned brown and subsequently died.

In an attempt to commercially release a glyphosate-resistant variety in Argentina, an in depth study on transgenic sugarcane events of elite variety, RA 87-3, expressing C4 *epsps* gene from *A*. *tumefaciens* that confers glyphosate resistance was conducted. To evaluate the potential impact on different agricultural systems and food safety necessary for commercial deregulation of any transgenic event in Argentina, several health and environmental regulatory studies were carried out (Noguera et al., 2015; Perera et al., 2020; Enrique et al., 2021; Ostengo et al., 2021). These studies demonstrated the feasibility to produce transgenic varieties indistinguishable from their parental genotype regarding physiological, agronomical and industrial characters. Another important discovery in this study was the importance of conducting a genetic analysis by employing molecular markers to find events with no or very low levels of genetic rearrangements after tissue culture regeneration after biolistic transformation of embryonic calli. After official presentation of a selected event to the National Advisory Commission on Agricultural Biotechnology (CONABIA), National Food Safety and Quality Service (SENASA) and the Secretariat of Agricultural Markets all three evaluation committees gave their approval for a commercial release in late 2015 (Noguera et al., 2019). Nevertheless, the final approval from the Ministry of Agriculture, Livestock and Fisheries for free commercial production remains pending due to concerns regarding exportation of sugar produced from a transgenic event. It is important to notice that this experience and interaction with regulatory agencies favored the generation of new regulations for the treatment of an agamic propagated crop such as sugar cane. Argentina’s regulatory system for evaluating transgenic crops has served as a reference for many countries (Lewi and Vicién, 2020). New regulations and assessment criteria have been developed for special cases like gene stacking, RNAi, and New Breeding technologies (NBT) where the aim has always been on how to gather and evaluate data in the most efficient possible way and only require information that is really needed to make a regulatory decision on biosafety (Lema, 2014). As part of this strategy an important change in requirements for evaluating vegetative propagated crops with polyploidy genomes such as sugarcane and potato was implemented in 2013, where a fast track evaluation of new events containing equal or similar gene constructs to previous evaluated events with favorable opinions was implemented^2^. This resolution permits a much faster approval of new sugarcane varieties with the same gene or similar gene construct regardless of the transgene position in the plant genome. This is of extreme importance as introgression of a transgene by backcrossing is impossible in sugarcane, leaving only two options to produce transgenic varieties, either by forward breeding or direct transformation of elite varieties. Both strategies are extremely time-consuming and many times when a transgenic variety is ready for commercial release the technology (parental variety) is obsolete. With this decision, the production of new varieties with characters of high interest can be achieved within a reasonable time frame and at much lesser cost. This same policy has later been implemented by Regulatory Agencies of Canada, United States and Brazil (Beker et al., 2016).

Nowadays, the majority of commercially grown genetically modified (GM) crops harbor genes related to both insect and herbicide resistance, which are both valuable characteristics in production of many crops, such as corn, soybean and cotton. The first sugarcane events expressing both traits were published by Wang et al. (2017b) demonstrating transgenic sugarcane harboring the C4 *epsps* gene for glyphosate resistance and *cry1Ab* for pest resistance. Five single-copy and ELISA-positive transgenic lines were tested under laboratory and field conditions to determine their agronomic and industrial traits and their resistance to insects and herbicides. Results showed that these transgenic lines showed strong insect resistance and glyphosate resistance under both growing conditions. However, in field conditions most of the transgenic plants presented poor agronomic and industrial characteristics compared to the non-transformed control plants (Wang et al., 2017b).

Recently gene editing technology was applied for the first time to confer herbicide tolerance in sugarcane. More details on gene editing technologies will be given in the corresponding section of this review; but in brief, CRISPR/Cas9 was used for co-editing mutations of multiple alleles of the acetolactate synthase (*ALS*) gene (Tufan Oz et al., 2021). The ALS enzyme catalyzes the biosynthesis of essential branched-chain amino acids (Smith et al., 1989) and wild-type alleles are strongly inhibited by several herbicides, such as sulfonylureas, imidazolinones, triazolopyrimidines, pyrimidinyloxybenzoates, and sulfanilamide-carbonyl-thiazolidinones.

It must be highlighted that further efforts are urgently needed to create more environmental-friendly herbicide resistant crops to ensure a more sustainable crop production and safeguard environmental quality by reducing the demand for harmful weed killing chemicals (Mulwa and Mwanza, 2006), a need that has recently increased dramatically with the negative effects on the environment caused by the excessive use of glyphosate.

## Abiotic Stress Tolerance

Drought, salinity, low and high temperatures and low soil fertility are all important abiotic factors negatively affecting sugarcane production in many production areas (Cursi et al., 2021b). Of these, drought is the single most important stress affecting sugarcane productivity in almost all production areas worldwide and therefore the development of more water deficit tolerant cultivars is of great interest to all sugarcane producing countries (Wang et al., 2003; Rampino et al., 2006; Ferreira et al., 2017). Sugarcane is a relatively water-demanding crop that produces 8–12 tons of sugarcane per one million liters of irrigation water (Kingston, 1994). The high water demand means that even relatively moderate water deficits can lead to productivity losses of up to 50–60% (Robertson et al., 1999; Ramesh, 2000; Basnayake et al., 2012; Lakshmanan and Robinson, 2013; Gentile et al., 2015). For this reason, production areas are mostly concentrated in regions with a favorable rain regime for sugarcane growth and development (Moreira, 2007), while in areas with less precipitation, additional or full irrigation is required (Walter et al., 2014).

Biotechnology and molecular breeding techniques could be helpful tools to enhance sugarcane tolerance to drought stress but despite increased availability of such technology and advancements in our understanding of plant water stress responses, genetically engineering crops for improved drought tolerance remains a vital challenge (Wang et al., 2003; Hu and Xiong, 2014). The complexity of plant responses to water deficit, and the arduous task of identifying and exploiting global effect genes and alleles and their associated traits, for developing more drought-tolerant varieties suitable for commercial crop production are difficult to overcome (Tardieu, 2012; Cominelli et al., 2013). In Table 5, an overview of various transgenic strategies to produce more drought tolerant sugarcane varieties is provided. In all of them, the selected gene to be expressed has been associated with plant water stress responses or shown to confer water stress tolerance in other species (Zhang et al., 2006; Reis et al., 2014; Augustine et al., 2015a; Ramiro et al., 2016). In this context, a transgenic sugarcane genotype expressing a bacterial gene encoding a choline dehydrogenase developed by Persero (PT Perkebunan Nusantara XI) probably becomes the first commercially released drought-tolerant transgenic sugarcane in the world^3^. This enzyme is involved in the synthesis of glycine betaine, a known osmoregulator that helps maintain water potential, protect cellular organization and biological functions during cellular dehydration (Sugiharto, 2018).

**TABLE 5:** Abiotic stress tolerance studies in transgenic sugarcane.

Abiotic stress	Promoter	Candidate Gene	Gene function	Transformation method	Variety	Stress time (days)	Experimental conditions	References
Drought	p35S enhanced	*Tsase*	Biomolecules stabilization	*Agrobacterium*	ROC10	15	G/F	Zhang et al., 2006
Cold	pCOR15a inducible	*ipt*	Cytoquinin synthesis	Biolistic	RB855536	7	G	Belintani et al., 2012
Drought	p35S	*AVP1*	Osmotic regulation	*Agrobacterium*	CP-77-400	15	G	Kumar et al., 2014
Drought	pRab17	*DREB2A CA*	Gene regulation	Biolistic	RB855156	6	G	Reis et al., 2014
Salinity	*pAIPC inducible*	*P5CS*	Proline synthesis	Biolistic	RB855156	28	G	Guerzoni et al., 2014
Drought/Salinity	pUBI	*PDH45/DREB2*	Nucleic acids metabolism; gene regulation	*Agrobacterium*/biobalistic	Co 86032	10	G	Augustine et al., 2015b
Drought/Salinity	pUBI	*HSP70*	Cellular components; stabilization	*Agrobacterium*	Co 86032	10	G	Augustine et al., 2015a
Drought	pUBI	*BI-1*	Program cell death regulation	Biolistic	RB835089	21	G	Ramiro et al. (2016)
Drought	p35S enhanced	*AVP1*	Osmotic regulation	Biolistic	CSSG-668	180	G	Raza et al. (2016)
Drought	pUBI	*SoP5CS*	Proline synthesis	*Agrobacterium*	Guitang 21	3	G	Li et al., 2018
Salinity	pUBI	*EaGly III*	Reduce oxidative damage	Biolistic	Co 86032	15	G	Mohanan et al., 2021
Drought	pUBI	*AtBBX29*	Gene regulation	Biolistic	NCo310	21	G	Mbambalala et al., 2021
Drought	p35S	*TERF1*	Gene regulation	*Agrobacterium*	XintaitangR22	21	G	Rahman et al., 2021
Cold	pUBI	*SoTUA*	α-tubulin synthesis	*Agrobacterium*	ROC22	10	G	Chen et al., 2021

*G, greenhouse; F, field.*

Regulatory genes like transcription factors (TFs) responsible for the induction of water stress-induced genes, are promising candidates to develop plants more tolerant to water deficit (Reis et al., 2014). Among the large group of TFs found in plants, the COR/DREB family constitutes the first group of transcription factors that were associated with induced abiotic stress gene regulation (Moran et al., 1994) and several members from this family of TFs, from different plant species, have successfully been employed in generating more drought tolerant sugarcane. The overexpression of *AtDREB2A* enhanced drought tolerance in sugarcane as demonstrated by a higher relative water content (RWC), photosynthetic rate, sucrose content and bud sprouting in greenhouse plants exposed to drought stress (Reis et al., 2014). Interestingly, no negative effect on biomass production was observed in these transgenic plants, a drawback observed in many other studies where a transcription factor is constitutively or ectopically expressed. In a similar study, the overexpression of the *Erianthus arundinaceus DREB2* gene generated improved tolerance to drought as demonstrated by reduced membrane damage, higher photosynthesis rates and higher gene expression levels of known stress-induced genes in transgenic plants exposed to stress, while an increased salinity tolerance was shown in leaf disk senescence and bud sprout assays comparing transgenic tissue with non-transformed parental plants. When co-transformed with a plant DNA helicase gene, *PDH45, and EaDREB2 gene*, transgenic plants showed a greater level of salinity tolerance when compared to single gene-transgenics of both these genes (Augustine et al., 2015b).

Heat shock proteins (HSPs) are known to play a major role in abiotic stress tolerance mechanisms in plants and other organisms. Sugarcane plants overexpressing an *E. arundinaceus HSP*70 gene, exhibited significantly higher stress-induced gene expression, cell membrane thermostability, relative water content, gas exchange parameters, chlorophyll content and photosynthetic efficiency (Augustine et al., 2015a). These results suggest that EaHSP70 plays a significant protective role in plants exposed to drought and salinity and the potential use of this gene in genetic engineering strategies to produce sugarcane plants with improved drought and salt tolerance.

B-box proteins mediate transcriptional regulations and protein–protein interactions in cellular signaling processes. B-box proteins thereby play an important role in coordinating physiological and biochemical pathway fluxes and are good targets for controlling stress responses in plants. Mbambalala et al. (2021) reported that the overexpression of *AtBBX*29 in sugarcane improved drought tolerance by maintaining a higher rate of photosynthesis and by enhancing antioxidant and osmolyte accumulation during stress. Another study, using a regulatory gene, revealed that overexpression of the tomato ethylene responsive factor (*TERF1)* in sugarcane conferred improved drought tolerance through increased accumulation of proline, soluble sugars and glycine betaine, reduced production of reactive oxygen species and reduced malondialdehyde (MDA) accumulation, which possibly resulted from activation of expression of stress-related genes by *TERF1* in plants exposed to stress (Rahman et al., 2021).

The *Arabidopsis* H^+^-PPase (*AVP*1) gene encodes a vacuolar membrane protein capable of increasing vacuolar solute content by active H^+^ uptake from the cytoplasm into the vacuoles. The *AVP*1 overexpression in transgenic sugarcane improved both drought and salt tolerance (Kumar et al., 2014; Raza et al., 2016) as demonstrated by increased RWC and maintained water, osmotic and turgor potential in leaves of transformed lines. In addition, transgenic plants showed increased size, length and root biomass after stress treatment.

The BAX subfamily stands out among the proteins that regulate the induction of reactive oxygen species (ROS)-signaling (Watanabe and Lam, 2008). The overexpression of a BAX inhibitor from *A. thaliana* (*BI*-1) enhanced tolerance to water deficit in sugarcane plants by suppressing endoplasmic reticulum-stress-induced plant cell death (Ramiro et al., 2016).

Sugarcane plants submitted to salt stress accumulate compatible solutes, such as the amino acid proline, which may counteract effects of salt accumulation in the vacuole, scavenge ROS and preserve cellular functions. The overexpression of two genes involved in proline synthesis, *P5CS* and *SoP5CS*, enhanced tolerance to salinity and drought in transgenic sugarcane (Guerzoni et al., 2014; Li et al., 2018).

Methylglyoxal (MG) is a highly cytotoxic metabolite formed as a result of growth under abiotic stresses in higher plants (Kaur et al., 2014). However, plants have developed an efficient tolerance mechanism to remove an increased accumulation of toxic metabolites, namely ROS scavenging enzymes of the glyoxalase pathway [glyoxalase I (Gly I), glyoxalase II (Gly II) and glyoxalase III (Gly III)]. Mohanan et al. (2021) overexpressed the *EaGly III* gene in sugarcane obtaining transgenic lines that exhibited significantly higher water status, gas exchange parameters, chlorophyll, carotenoid, and proline content, total soluble sugars, superoxide dismutase and peroxidase activity compared to non-transformed parental plants under salinity stress.

Finally, several studies concerning transgenic sugarcane in response to low temperature stress are also detailed in Table 4. As a tropical plant species, sugarcane is very sensitive to exposure to chilling (low but non-freezing temperatures) and freezing, resulting in lower yields and reduced industrial quality of plants. Belintani et al. (2012) overexpressed a bacterial gene encoding the enzyme isopentenyl-transferase (*ipt*) under control of the *A*. *thaliana* cold-inducible promoter At*COR15*a. Transgenic plants expressing *ipt* showed higher leaf chlorophyll content, reduced MDA concentration and electrolyte leakage than non-transformed parental plants when subjected to freezing temperature. In another approach, the overexpression of an α-tubulin gene (*TUA*) in the cold susceptible variety ROC22 showed a higher concentration of total soluble proteins and sugars, increased peroxidase (POD) activity and lower MDA content than plants of the parental variety when exposed to chilling (Chen et al., 2021).

Although many different strategies (genes) have been employed to genetically engineer improved abiotic stress tolerance in sugarcane, there are still many challenges to overcome before commercially useful water stress, salinity and/or cold tolerant varieties are released. As seen from almost all studies described above there are very few field studies conducted and more information on the behavior of a transgenic event under natural growing and production conditions is required to correctly assess a possible effect on stress tolerance. Another area for future research is the stacking of genes involved in abiotic stress tolerance to evaluate the possibility to complement protective mechanisms effects and even search for possible synergistic effects. However, many of these studies are demonstrating promising results and it is therefore more than likely that commercial events with improved abiotic stress tolerance will be released in a nearby future.

## Industrial Traits

In the above sections we have described a great variety of transgenic sugarcane designed to improve agronomic traits of this crop such as disease, pest, herbicide and abiotic stress management. There are; however, strategies to improve industrial traits as well. One area that has generated a lot of interest has been the modification of sugar metabolism to increase sugar accumulation but with relatively little success (Wu and Birch, 2007; Groenewald and Botha, 2008; van der Merwe et al., 2010; Hamerli and Birch, 2011). In parallel, attention has been focused on improving bioenergy and biofuel production of sugarcane where strategies to alter the lignin composition of the plant to facilitate saccharification for production of second generation bioethanol (Jung et al., 2013; Kannan et al., 2018) and the alteration of the carbon-partitioning balance to produce triacylglycerols in vegetative tissues of sugarcane for biodiesel production (Zale et al., 2016), have been reported. Another industrial area of interest is the production of bioplastics like polyhydroxyalkanoates (PHAs). PHAs are biodegradable polymers produced by a wide-range of bacteria and strategies to produce these polymers in sugarcane such as polyhydroxybutrate (PHB) have been conducted (Petrasovits et al., 2007, 2012). Finally, the use of sugarcane as a platform for producing high value compounds or pharmaceuticals due to its high biomass and fast growth is a more than likely future new application although no direct approach of such a strategy has been published (Appunu et al., 2017).

## Genome Editing

Genome editing (GE) is a technology to engineer genetic modifications in which DNA is inserted, deleted or replaced within a specific sequence of the genome of an organism by using engineered nucleases. Nowadays there are four families of engineered nucleases available: meganucleases (MN), Zinc-finger nucleases (ZFNs), Transcription Activator-like Effector Nucleases (TALENs) and the Clustered Regularly Interspaced Short Palindromic Repeat (CRISPR)-Associated Nuclease 9 (Cas9) technology (Mohan, 2016). GE has found its application in a wide range of economically important crops providing higher yields, increased nutritional quality, weed protection and improved abiotic and biotic stress tolerance/resistance (diseases and pests) (Rahman et al., 2019). In addition, compared to other genetic manipulation methods, GE is considered a user-friendly tool for its ability to generate non-transgenic genome edited crop plants (Rahman et al., 2019).

In respect to GE technology applied to sugarcane genome modification, there are only a few successful studies published, which is understandable due to the very complex genome of this species and the lack of a sequenced reference genome. The first study was published by Jung and Altpeter (2016) who used TALEN to modify the cell wall composition by reducing lignin content in an attempt to improve saccharification efficiency and to facilitate and reduce costs for production of lignocellulosic bioethanol. Field-grown TALEN-mediated mutants of the caffeic acid *O*-methyltransferase gene showed a significant reduction in total lignin as well as a change in lignin composition, which resulted in improved saccharification efficiency (up to 43.8%) (Kannan et al., 2018).

Recently, Eid et al. (2021) reported efficient multi-allelic editing in sugarcane of the magnesium chelatase I subunit (*MgCh*), a key enzyme for chlorophyll biosynthesis. CRISPR/Cas9-mediated targeted co-mutagenesis of 49 copies/alleles of *MgCh* was confirmed *via* Sanger sequencing and resulted in severely reduced chlorophyll content. The successful application of this method will facilitate the establishment of genome editing protocols for recalcitrant plant species and support further optimization. Subsequently, Tufan Oz et al. (2021) demonstrated the precise co-editing of multiple alleles *via* template-mediated and homology directed repair (HDR) of DNA double-strand breaks, induced by the programmable nuclease CRISPR/Cas9. The evaluation resulted in co-editing of up to three acetolactate synthase (ALS) copies/alleles involved in herbicide resistance.

Using a GE approach in sugarcane today is a great challenge due to the high polyploidy, genome size, low transformation efficiency and transgene silencing frequencies that has been reported for this plant species (Mohan, 2016). Nevertheless, when more genome sequence information is available, more GE strategies to design specific guided RNAs for targeting specific genes should be readily applicable in sugarcane. Currently, plants edited by GE-technology are classified as GM in some countries, but not in others. In the latter case, the costs and efforts for a commercial release are greatly reduced, and hopefully more countries will soon facilitate deregulation of varieties produced using GE technology.

## Conclusion and Future Perspectives

The use of genetic transformation in sugar cane has advanced steadily over the years, demonstrating that the crop can be successfully genetically engineered, incorporating characteristics of interest. Through the use of efficient genetic transformation methods and different screening strategies, events with low copy number and high levels of stable expression of transgenes can readily be obtained.

Genetic engineering has made possible the development of transgenic sugarcane events that express key characteristics such as resistance to herbicides, diseases, pests and tolerance to abiotic stresses, many of which have shown excellent performances both in greenhouse experiments and in field trials. But despite of this, very few breeding programs in the world have presented transgenic varieties for a commercial release. Although there are currently numerous developments of transgenic sugarcane lines in advanced stages of evaluation in different countries, the commercial release of transgenic sugarcane lags behind many other important crops such as soybean, corn, cotton and canola, even though the food product for consumption from sugarcane does not contain DNA.

This can to a certain extent, be explained by the arduous and costly process to produce a transgenic sugarcane variety due to the extremely complex genetics of this crop, where every new variety must go through the complete transformation and deregulation process or be part of a forward breeding strategy, as introgression by backcrossing is not feasible. It is therefore of utmost importance to shorten and make more efficient deregulation times, in order to allow transgenic varieties to be released more quickly to the market, and to prevent the genotypes used becoming obsolete. An important step in this direction is the decision in Argentina and other countries to allow for a more rapid release of a second variety of the same crop with the same or very similar genetic construction as a previously released event. In addition, the recent marketing approvals for transgenic sugarcane events in Indonesia and Brazil are expected to provide a boost to other sugarcane producing countries, such as India, China, Thailand, the United States and Argentina, to continue research and develop new transgenic varieties that can contribute to effective solutions to the challenges facing the crop in different agroecological settings where it is grown. Furthermore, under the new paradigm of sustainable agriculture, sugar cane plays a fundamental role both in food production and as a high-performing renewable energy source.

In addition, a number of sub- and residual products from the sugar-ethanol industry can be used as important bases for new products under the concept of biorefinery, properties that can be improved and expanded to new applications by producing specially designed transgenic varieties with new properties. But for this to be viable times and cost for commercial release must be considerably reduced.

Finally, gene editing techniques based on CRISPR-Cas9 and others, already applied in crops such as rice and wheat, are still in the initial stages of implementation in sugarcane. This delay can be attributed to the genetic complexity of the crop and to a lower availability of genomic information, essential to determine *a priori* which sequences to be modified and the possible effect this will have on the rest of the genome. Given that gene editing could dramatically reduce the time and costs required for commercial deregulation, the use of this technology is expected to increase in the foreseeable future. It is important to highlight that to guarantee the success of this strategy in complex polyploid genomes, it is essential to have a robust genetic transformation method that allows the introduction of the GE system in the different genetic backgrounds of elite sugarcane varieties obtained through classical breeding programs.

## Author Contributions

FB, RE, MP, JR, AC, AN, and BW have made an equal and important contribution to the manuscript, each one in their area of expertise. BW performed the final overall revision.

## Conflict of Interest

The authors declare that the research was conducted in the absence of any commercial or financial relationships that could be construed as a potential conflict of interest.

## Publisher’s Note

All claims expressed in this article are solely those of the authors and do not necessarily represent those of their affiliated organizations, or those of the publisher, the editors and the reviewers. Any product that may be evaluated in this article, or claim that may be made by its manufacturer, is not guaranteed or endorsed by the publisher.

## Footnotes

1
www.cenicaña.org

2
https://www.argentina.gob.ar/normativa/nacional/resolución-318-2013-218394/texto

3
https://www.isaaa.org/gmapprovaldatabase/event/default.asp?EventID=340&Event=NXI-1T
